# Use of natural deep eutectic systems as new cryoprotectant agents in the vitrification of mammalian cells

**DOI:** 10.1038/s41598-022-12365-4

**Published:** 2022-05-16

**Authors:** Ana Rita Jesus, Ana Rita C. Duarte, Alexandre Paiva

**Affiliations:** LAQV-REQUIMTE, Campus da Caparica, Monte da Caparica, 2825-149 Caparica, Portugal

**Keywords:** Biotechnology, Chemical biology

## Abstract

In this work we present the potential of Natural Deep Eutectic Systems (NADES) as new vitrification media for the cryopreservation of mammalian cells. Several NADES composed of natural metabolites were prepared and tested as CPAs in two cell lines, L929 and HacaT cells. After the harvesting, cells were mixed with the eutectic systems, and frozen directly into liquid nitrogen to achieve a vitreous state. Then, the cells were thawed and it was observed that NADES were able to exert a significant cryoprotective effect in L929 cells, when compared with DMSO or in the absence of a CPA. For HacaT cells, only a eutectic system showed a slightly improvement in cell survival, while DMSO caused complete cell death. Moreover, the thermal behaviour of the best systems was studied for further understanding the protective properties of NADES as CPAs, and have shown a significant difference in terms of *T*_m_ and *T*_c_ when compared with DMSO and water. Additionally, the results obtained showed that NADES can be maintained in the growth media after the thawing step, without compromising cell viability. In summary, we have shown the great potential of NADES to be used as CPAs for the cryopreservation of different cell types, using the vitrification method.

## Introduction

Long-term preservation of biological material requires the cessation of the biological reactions, and is typically done at sub-zero temperatures. Then, the formation of ice inevitable occurs, often with deadly consequences. The inhibition of ice formation, particularly intracellular ice, is known to be the key factor for a successful cryopreservation^1–3^. This inhibition can only be achieved when specific solutes, called cryoprotectant agents (CPAs), are added to the cells. Then, two different methods can be used to freeze cells. In the first, the slow-cooling method, is given enough time for the cell to dehydrate as a result of extracellular ice formation and subsequent exosmosis during the cooling process. Then, an increase of the concentration of CPA in the cytoplasm occurs which prevent the lethal intracellular ice formation^3,4^.

In the second method a high concentration of CPA is used, and the solution of cells tuns into non-crystalline or amorphous solid known as glass, reason why this method is called vitrification^3,5,6^.

During the freezing process, heterogeneous or homogeneous nucleation may occur. Homogeneous nucleation occurs when self-aggregation of water molecules happens but it only occurs when the temperature reaches very low temperatures, e.g., − 39 °C in pure water. On the other hand, heterogeneous nucleation occurs when the water molecules freeze on a foreign particle suspended in the solution. The prevention of heterogeneous nucleation during the cooling process is the key factor to achieve the vitreous state^4,7^. However, when dilute solutions are being cooled, the inhibition of homogenous nucleation is more difficult to accomplish. But, an increase in the concentration of a CPA in the solution can significantly decrease the homogeneous nucleation temperature (T_hom_) and at a certain concentration of CPA it is possible to decrease T_hom_ below the glass transition temperature (T_g_). Unfortunately, high concentrations of CPA are usually highly toxic for biological systems. Therefore, it is necessary to find a specific ratio between concentration, toxicity and depression in T_hom_ to achieve optimal conditions for a vitrification process^8,9^.

Cryoprotectant agents (CPAs) are classified as penetrating (small molecular weight) and non-penetrating (high molecular weight) CPAs. Penetrating CPAs reduce dehydration avoiding ice growth. Some examples are ethylene glycol (EG), dimethyl sulfoxide (DMSO), propylene glycol (PG), and glycerol^8,10^. On the other hand, non-penetrating CPAs remain in the extracellular compartment without penetrating the cell membrane, promoting glass formation. Sugars (sucrose and trehalose) and high molecular weight polymers (PVP and Picoll) are some reported examples of non-permeating CPAs^9–11^. It has been reported that the combination of non-permeating and permeating CPAs was able to improve post-thaw viability and functionalities of vitrified mammalian tissues^10^.

The toxicity of the common CPAs, such as DMSO, combined with the low post-thaw viability of cells led the researchers to search for alternatives to achieve improved cryopreservation results.

Natural deep eutectic systems (NADES) have been applied in a variety of applications^12–16^. These systems result from the combination of natural primary metabolites, such as sugars, amino acids, organics and choline derivatives, in specific molar ratios. NADES present a high melting point depression, when compared with the individual components, hence being liquids at or near room temperature^12^. These systems present a high viscosity that can the tailored by changing the water content, which also allows to decrease their toxicity^17^.

In terms of thermal behaviour, NADES have already showed thermal stability at a wide range of temperatures, even far below 0 ºC. This strongly supports the hypothesis proposed by Dai et al.^18^ regarding the role of NADES in cold resistance. Furthermore, Craveiro et al.^19^ showed the relationship between the water content of NADES and the decrease on the glass transition temperature of the mixture. Additionally, Castro et al.^20^ showed that the eutectic system trehalose:glycerol (1:30) was able to promote water vitrification in a large range of temperatures, and therefore showing its potential to be used as CPA.

Saccharides, such as sugars (e.g., trehalose^11,21^, sucrose^22^, glucose^23^) and sugar alcohols (e.g. mannitol^24^) have been showed to be excellent candidates for cryopreservation of biological materials. However, some of them remain on the outside of the cells since there are no specific transporters for them in mammalian cells. However, their cryoprotective action alone is insufficient to guarantee the preservation of structural and functional integrity of cells. Therefore, they are generally combined with permeating agents such as DMSO or glycerol^25^.

In our previous report^26^ we have prepared a library of NADES composed by natural metabolites, sugars, amino acids, and choline derivatives and showed that they were able to successfully cryopreserve two different types of cells, L929 and HacaT, when used at 10% (w/v). what we propose here is to use NADES at high concentration as CPA for the vitrification of mammalian cells.

## Results

### Preparation of NADES

NADES presented in Table 1 were prepared as described previously by our group^26^. These systems are composed by natural metabolites, such as trehalose, glucose, proline and sorbitol. For the majority of the systems, water was required to ensure the formation of the eutectic mixture which was previously confirmed by polarized optical microscopy (POM) and nuclear magnetic resonance (NMR)^26^.

**Table 1:** Composition of NADES used in this study.

NADES	Components				Molar ratio	Water content (%wt)
	A	B	C	D		
TreGluSorW	Trehalose	Glucose	Sorbitol	Water	1:2:1:10	17.2 ± 0.6
GluProGlyW	Glucose	Proline	Glycerol	Water	3:5:3:21	21.5 ± 1.3
GlyGluSorW	Glycerol	Glucose	Sorbitol	Water	1:1:1:3	11.9 ± 0.6
BetGlySucW	Betaine	Glycerol	Sucrose	Water	2:3:1:5	10.3 ± 0.4
BetTreW	Betaine	Trehalose	Water		4:1:12	22.0 ± 0.3
GlySucSorW	Glycerol	Sucrose	Sorbitol	Water	2:1:2:10	16.3 ± 0.5
BetTreGlyW	Betaine	Glycerol	Trehalose	Water	2:3:1:7	12.6 ± 0.3
BetSucProW	Betaine	Sucrose	Proline	Water	5:2:2:21	20.2 ± 0.5
GlyGlu	Glycerol	Glucose		Water	4:1:1	-
GlyTreSorW	Glycerol	Trehalose	Sorbitol	Water	2:1:2:12	20.4 ± 0.8
FruGluTreW	Fructose	Glucose	Trehalose	Water	1:1:1:15	27.1 ± 0.8

### Freezing L929 and HacaT Cells using NADES as CPA

In this study two different cell lines were used to study the ability of NADES to be used in vitrification media as cryoprotectant agents (CPA). Therefore, L929 and HacaT cells were mixed with 50% (w/v) of different NADES, transferred to cryovials and frozen directly in liquid nitrogen. In a first stage, cells were also frozen without a CPA and with 50% (v/v) of DMSO, for comparison. The freezing process only took a few seconds until the samples were completely frozen. The vials were kept in liquid nitrogen for additional 5 min, and then transferred to a − 80 °C freezer for two weeks prior the post-thawing assays.

### Post-thawing viability of L929 and HacaT cells

**Figure 1:**
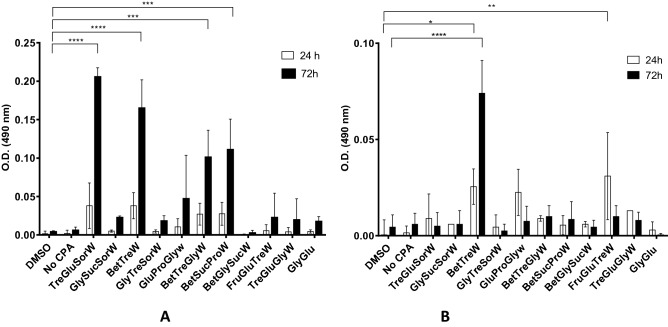
Initial screening of the post-thawing viability after vitrification in liquid nitrogen with either DMSO, no CPA or NADES. (**A**) in L929 cells. Data represent means ± SD (n = 2). Statistically significant differences are represented in asterisks: ***p < 0.001 and ****p = 0.0001, two-way ANOVA. The absence of asterisks means that there are no significant differences when compared with values of DMSO at the corresponding time points. (**B**) in HacaT cells. Statistically significant differences were determined by Turkey’s multiple comparisons test and are represented in asterisks: *p < 0.05, and **p < 0.005; and ****p = 0.0001, two-way ANOVA. The absence of asterisks means that there are no significant differences when compared with values of DMSO at the corresponding time points.

Three vials frozen with each formulation were rapidly thawed at 37 °C (no more than 1 min), diluted and centrifuged for counting. Cells were then plated at 1.5 × 10^5^ cells/mL and incubated for 24 h-72 h in the presence of 5% (w/v) of NADES or 5% (v/v) of DMSO. Cell viability was determined at 24 h, 48 h, and 72 h after the thawing (Fig. 1). It is visible that NADES worked fairly better in L929 cells than in HacaT cells. Most NADES successfully cryopreserved L929 cells, while only the eutectic system BetTreW afforded promising results in HacaT cells. Moreover, freezing the cells without any CPA resulted in complete cell death. The presence of NADES during the post-thawing studies did not compromise cell viability, suggesting that the systems do not need to be removed after thawing the cells and therefore can be transferred directly to the culture flask. Moreover, after 24 h of the thawing the culture media was changed and fresh complete media was added to the cells.

After this initial screening, both cell lines were frozen with the best NADES for further studies, namely, thermal behaviour, morphology, and proliferation studies. For L929 cells the systems TreGluSorW, BetTreW, and BetSucProW were selected, and for HacaT cells only the system BetTreW was used for the subsequent studies.

### Thermal behaviour by calorimetry/POM

For a better understanding on the thermal behaviour of the systems that allowed a higher rate of cell survival after thawing when used at 50% (w/v) as CPA were selected and their aqueous mixtures were analysed by calorimetry and the physical changes were observed, simultaneously, by polarized optical microscopy (POM). The heat flow *vs* temperature curves of these aqueous mixtures are presented in Supplementary Information (Figure S1). In Table 2, are listed the values of *T*_c_ and *T*_m_ of the NADES aqueous mixtures, DMSO and pure water.

**Table 2:** Melting temperatures for the aqueous mixtures of BetSucProW, BetTreW, TreGluSorW, DMSO and distilled water. All these values refer to the ones obtained at onset temperature.

50% (w/v) aqueous solution	*T*_c_ (ºC)	*T*_m_ (ºC)
BetSucProW	− 26.6	− 13.2
BetTreW	− 32.3	− 11.7
TreGluSorW	− 31.0	− 14.6
DMSO*	–	–
100% Distilled H_2_O	− 23.7	0.6

*For DMSO 50% v/v solutions were used.

From the results presented in Table 2, and Figures S1, it is possible to see that the aqueous mixture of DMSO (50% v/v) does not freeze in this temperature range, which can explain the very low viability of both cell lines when frozen with DMSO at 50% (w/v). If the water does not freeze then the DMSO present in the solution will cause the death of cells due to its toxicity.

Moreover, both crystallization and melting temperatures of aqueous solutions of NADES at 50% (w/v) are much different than those of pure distilled water. Due to equipment limitation lower temperatures than − 90 °C could not be achieved. We do believe that DMSO vitrifies at much lower temperatures than − 90 °C or that state can be achieved once the sample is placed directly in liquid nitrogen which presents a much higher cooling rate than our experiment, but that fact contributes to a higher toxicity of this CPA in these conditions. Additionally, it is possible to observe in the physical changes’ images that when water freezes (Figure S1 F) there is a generation of interference effects in the polarized optical microscope which results in differences in colours, showing bright crystals. However, when the aqueous solutions of NADES freeze, the shape of the crystals is completely different (Figure S1 A-D), showing a black image, which confirms the amorphous state of 50% (w/v) aqueous solutions of NADES at the corresponding T_c_.

### Morphology and proliferation studies

**Figure 2:**
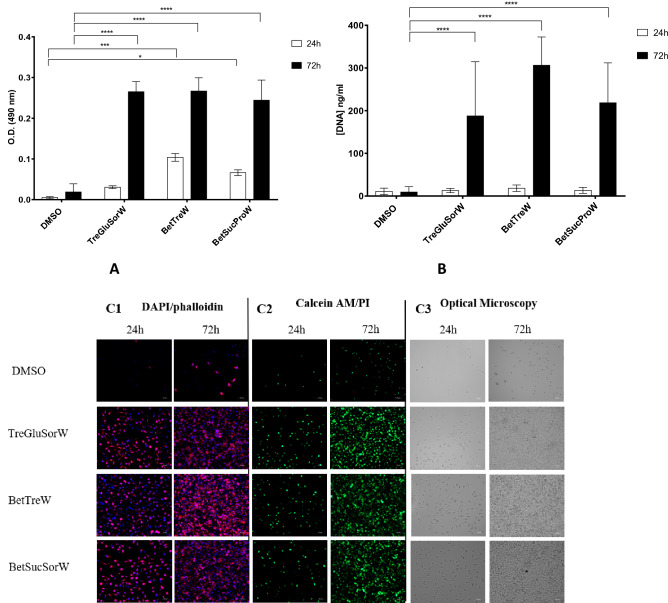
Assessment of the post-thawing recovery of L929 cells. MTS assay (**A**) and DNA Assay (**B**) after 24 h, 48 h, and 72 h of culture.Data represent means ± SD (n = 3). Statistically significant differences were determined by Dunnett’s multiple comparisons test and are represented in asterisks: *p < 0.05; ***p < 0.005 and ****p = 0.0001, two-way ANOVA. The absence of asterisks means that there are no significant differences when compared with values of DMSO or other NADES at the corresponding time points. Post-thawing DAPI/Phalloidin assay (cells nuclei were stained blue by DAPI and F-actin filaments in red by phalloidin) (**C1**), Live/Dead fluorescence assay (living cells were stained green by calcein AM and dead cells red by PI) (**C2**) and Optical microscopy (**C3**) of L929 cells at 24 h and 72 h.

After thawing, L929 and HacaT were stained with DAPI/Phalloidin to observe the cell nuclei and the F-actin filaments, and the Live/Dead fluorescence assay was performed to observe the live and dead cells. The results presented in Fig. 2 show that NADES worked significantly better than DMSO on the cryopreservation of L929 cells. Complete confluency of L929 cells was observed after 3 days of the thawing.

**Figure 3:**
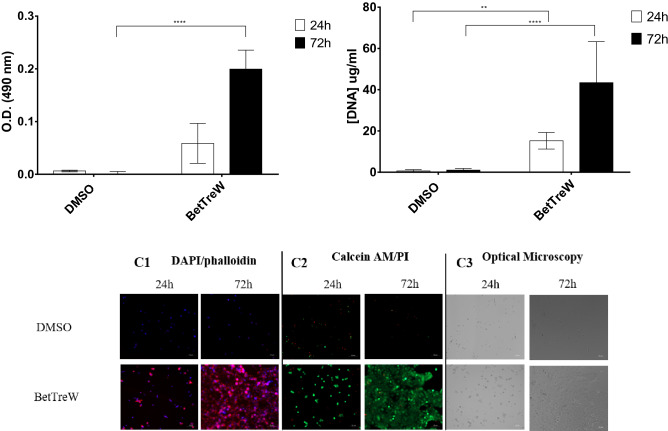
Assessment of the post-thawing recovery of HacaT cells. MTS assay (**A**) and DNA Assay (**B**) after 24 h, 48 h, and 72 h of culture. Data represent means ± SD (n = 3). Statistically significant differences were determined by Dunnett’s multiple comparisons test and are represented in asterisks: **p < 0.01; ***p < 0.05; and ****p = 0.0001, two-way ANOVA. The absence of asterisks means that there are no significant differences when compared with values of DMSO at the corresponding time points. Post-thawing DAPI/Phalloidin assay (cells nuclei were stained blue by DAPI and F-actin filaments in red by phalloidin) (**C1**), Live/Dead fluorescence assay (living cells were stained green by calcein AM and dead cells red by PI) (**C2**) and Optical microscopy (**C3**) of HacaT cells at 24 h and 72 h.

Although the system BetTreW presented lower efficacy towards HacaT cells than towards L929 cells, this the eutectic system was able to afford better results when compared with DMSO (Fig. 3). Nevertheless, it was observed that after 3 days several large colonies were observed in the wells of cells cryopreserved with BetTrehW, which was not observed for the DMSO.

Similar studied were carried out for passages P1 and P2 and the results showed normal cell morphology and high proliferation ratios in both cell lines (Figure S2 and S3).

## Discussion

The goal of this work was to find alternatives to DMSO for the cryopreservation, through the vitrification method, of mammalian cells and potentially for other cell types and tissues. In literature it is possible to find only a reports for the vitrification of mammalian cells, mostly using advanced technologies, such as electroporation^27^. Therefore, the results presented in this manuscript show natural cryoprotectants can be used as vitrification media, using a simple methodology, for numerous applications in cryobiology.

From literature, a permeating agent must ideally be present in a vitrification solution^28^. However, in this work we were able to show that this is not actually mandatory and that large molecules (non-penetrating) are, in fact, able to protect the cells during the vitrification process.

Our work focused on the use of NADES as CPA for the vitrification of mammalian cells. These NADES are mainly composed by primary metabolites commonly found in animals and organisms that live in extremely cold environments, as well as, common components of culture media, such as sugars, polyols, and amino acids (Table 1)^18^.

Most of the systems prepared contain the mono- and disaccharides, glucose, trehalose and sucrose which exert considerable osmotic effects as smaller non-permeating molecules, such as betaine and many amino acids. In fact, the incorporation of a small saccharide promoted the cell dehydration and its vitrification, reducing the concentration of intracellular cryoprotectant and consequently its toxicity, as observed during a study where sucrose alone was able to significantly decrease the toxicity of a vitrification solution for the cryopreservation of mouse embryos^29^.

Then, 50% (w/v) of NADES were used as CPA for the vitrification of mammalian cells. The reason of using 50% (w/v) instead of higher percentages of NADES is highly related to their high toxicity at those concentrations, as well as, their high viscosity which would difficult the diffusion process during freezing.

Vitrification is known to be achieved when the samples are frozen at freezing rates of 15,000–30,000 °C/min, generally, achieved by submersing them directly in liquid nitrogen. However, to the extent of our knowledge there are no reports using this method to vitrify mammalian cells in cryovials, as is usually performed using the slow-freezing method. Herein, we have shown that it is, in fact, possible to successfully cryopreserve mammalian cells in cryovials using the vitrification method, using NADES as CPA. For that, cryovials were immediately immerse in a container with liquid nitrogen before transferring them to the − 80 °C freezer. This method allowed the immediate freezing of the samples. Although requiring extra care during the handling of liquid nitrogen it is a simple methodology, not requiring any state-of-the-art equipment.

An initial screening was performed, in two cell lines, L929 and HacaT cells, to select the most promising systems (Fig. 2). From the results we have seen that for L929, NADES exerted a much more pronounced cryoprotective effect than DMSO. On the other hand, for HacaT cells only one eutectic system (BetTreW) showed promising results. However, HacaT cells frozen with 50% (v/v) DMSO were not able to survive in this method. In general, this method resulted in considerable better results for L929 cells, which is a more robust and resilient cell line. The results can be explained by the thermal behaviour of the aqueous mixtures of NADES and DMSO. While 50% (w/v) aqueous mixtures of NADES are able to freeze, although in an amorphous state, in the conditions reported in this study, the 50% (v/v) aqueous mixtures of DMSO cannot completely freeze in the same conditions. This fact contributes to cells to die due to the high toxicity of DMSO.

In this work, we have also shown that after thawing, the vitrification media does not need to be removed and a simple dilution is sufficient to reduce the toxicity of the CPAs. Then, cells can be directly cultured in T-flasks and/or plated which can be a major advantage because the centrifugation step or washing step to remove the CPA can cause a certain degree of cell death. This can be particularly relevant if this method is used during cell therapy or transplantation procedures.

The systems TreGluSorW, BetTreW and BetSucProW showed the most promising results towards L929 cells, while for HacaT, the system BetTreW was the only system showing favourable results. Hence, further studies were carried out with these systems, including thermal behaviour, morphology and proliferation studies. The thermal studies, using a DSC/POM equipment have shown that the 50% (v/v) aqueous solution of DMSO does not freeze in the experiment conditions, but the aqueous solutions of the selected NADES showed a significant decrease on the *T*_c_ and *T*_m_ when compared with pure distilled water. Additionally, the shape of the crystals is completely different between the NADES aqueous solutions and water. The major advantage of the method used herein is the fact that we used polarized light which helps to distinguish crystalline and amorphous states. In the case of NADES aqueous solutions we observed black images confirming the amorphous state of the samples, while with water bright and coloured crystals were observed.

The NADES studied in this work did not affect the morphology of the cells after freezing and thawing as showed by the morphology studies after 72 h of the thawing (Figs. 2C1 and 3C1). In terms of proliferation, we observed that NADES did not interfere with the normal proliferation of L929 cells but, on the other hand, DMSO significantly compromised the cell growth (Fig. 2A,C2). Furthermore, DMSO contributed to a very low DNA content while with NADES the complete opposite was observed, after 72 h of thawing.

Regarding the HacaT cells it was possible to see that the system BetTreW allowed a successful post-thawing viability, and after 72 h very large colonies were observed (Figs. 2A,C2) and after 5 days cells were completely confluent (not showed). In terms of DNA content, the same trend was observed.

Both L929 and HacaT that showed significant post-thawing survival were cultured up to 3 passages, and the same studies were conducted for different passages (P1 and P2). The results showed that after the first passage, cells presented high values of viability and an improvement of proliferation, suggesting that NADES do not affect cellular activity (Figure S2 and S3).

In summary, we have shown that NADES are an emerging class of CPA that can be used for the cryopreservation of mammalian cells using the vitrification method. Our study showed that the L929 cells were able to be cryopreserved in the presence of the eutectic systems TreGluSorW, BetTreW, and BetSucProW. Additionally, the system BetTreW was able to confer cryoprotective properties to HacaT resulting in a successful cryopreservation. Nevertheless, the possible combinations of primary metabolites able to be used as CPA are endless and therefore in further studies other systems might be studied. Furthermore, it is important to highlight that the major advantage of these new materials is the fact that they contribute for cell survival without their removal from the growth media.

The results presented herein show that NADES are potential CPA for the vitrification of other biological materials such as stem cells, sperm, embryos, tissues, etc.

## Methods

### Preparation of NADES

NADES (Table 1) were prepared by gently mixing the corresponding components as described elsewhere^26^. Briefly, the components of each eutectic system were mixed in their specific molar ratio, in a closed vial and heated (with stirring) at 60 °C or 40 °C, if amino acids are present, until the the mixture becomes a viscous transparent liquid.

### Cell culture

Biological studies^26^ were conducted in L929 (DSMZ—German Collection of Microorganisms and cell culture GmbH) and HacaT cell lines (DKFZ, Heidelberg) which are mouse fibroblasts of connective tissues and human keratinocytes, respectively. L929 cells were grown in Eagle’s Minimum Essential Medium (MEM, with 1.5 g/L sodium bicarbonate, non-essential amino acids, L-glutamine and sodium pyruvate, Corning), supplemented with 10% fetal bovine serum (FBS, Corning) and 1% penicillin–streptomycin (Corning). HaCaT cells were grown in Dulbelco’s Modified Eagle Medium (DMEM with 4,5 g/L glucose, L-glutamine and sodium pyruvate, Corning) supplemented with 10% fetal bovine serum (FBS, Corning) and 1% penicillin–streptomycin (Corning). Both cell lines were cultured in a humidified incubator at 37 °C, with 5% CO_2_.

### Cryopreservation protocol

The cryopreservation protocol was adapted from our previous work^26^. Briefly, after trypsinization, confluent cells were harvested and collected by centrifugation (200 g, 10 min). Then 1.5 × 10^6^ cells/mL were resuspended in freezing media (MEM + FBS, 70:30 for L929 cells and DMEM + FBS, 90:10, for HacaT cells) containing 50% (w/v) of NADES or 50% (v/v) DMSO, or no CPA. Then cryovials were immediately immersed in liquid nitrogen for a couple of second until complete freezing and the 5 additional minutes. Then, the vials were transferred to a − 80 °C freezer for two weeks prior the post-thawing assays.

### Thawing protocol

As previously described^26^ the cryovials were rapidly warmed up in a 37 °C water bath for 1 min (maximum), then rapidly transferred to a falcon tube containing complete media and centrifuged (200 g, 10 min). After discarding the supernatant solution, the cells were resuspended in complete media, and plated at 1.0 × 10^4^ cells/well in 96-well plates. Cell viability, proliferation and morphology was evaluated after 24, 48 and 72 h, and compared with those frozen using DMSO and without CPA. Cells were sub-cultured up to 3 passages.

### Cell viability assay

After 24 h, 48 h and 72 h of thawing, cell monolayers were washed with PBS and cell viability was evaluated using the CellTiter 96^®^ AQueous One Solution Cell Proliferation Assay (Promega), which is based on tetrazolium active component ((3-(4,5-dimethylthiazol-2-yl)-5-(3-carboxymethoxyphenyl)-2-(4-sulfophenyl)-2H-tetrazolium, MTS). The amount of formazan product was measured in a microplate reader (VICTOR Nivo™, PerkinElmer, USA) at 490 nm, as absorbance is directly proportional to the number of viable cells in culture. Cell viability was expressed as percentage of cells exposed to extracts *vs* control cells^26^.

### DSC/POM studies

The selected systems were used for DSC/POM studies. Solutions of 50% (v/v) of DMSO and 50% (w/v) of the selected NADES were prepared. Then, ca. 10 mg of each solution was placed in the sapphire crucible which was then placed into the cooling stage. Afterwards, a cooling run was carried out using the following settings: (1) 1 min at 25 °C; (2) cooling up to − 90 °C at 30 °C/min; (3) heating up to 40 °C at 10 °C/min; (4) cooling up to 25 °C at 10 °C/min. Liquid nitrogen speed was set on 80. At the same time of the DSC runs the physical changes were observed using a microscope equipped with a polarized light.

### DNA quantification

The Quant-IT PicoGreen dsDNA Assay Kit (Invitrogen Life Technologies, Scotland) was used for DNA quantification as previously described^26^ and according to manufacturer’s instructions. Briefly, 24 h after thawing the cells, DNA was extracted after osmotic and thermal shock. Triplicates were collected for each sample. The absorbance was read in a microplate reader (VICTOR Nivo™, PerkinElmer, USA), using 485 and 528 nm as excitations and emission wavelengths, respectively. The amount of DNA was calculated from a standard curve.

### Cell Morphology

Cells were fixed after 24 h, 48 h and 72 h post-thawing with 10% (v/v) formalin solution (neutral buffered, Sigma-Aldrich). Then, cells were stained with 4,6-diamidino-2-phenyindole (DAPI, Corning) and phalloidin–tetramethylrhodamine B isothiocyanate (phalloidin, Sigma-Aldrich) to visualize F-actin filaments and cell nuclei, respectively. Briefly, DAPI (2 µg/ml) and phalloidin (2.5 µg/mL) were added to each well and incubated for 30 min at room temperature and protected from light. After washing the cells with PBS, the morphologic changes were visualized in the dark using an inverted microscope (Axio Vert A1, Zeiss, Oberkochen, Germany)^26^.

### Live/dead assay

Live/dead assay was performed after thawing the cells to evaluate the post-thawing rate survival, using calcein AM/propidium iodide (PI) staining. At predetermined timepoints, cell monolayers were incubated for 20 min at 37 °C in the presence of calcein AM (0.25 µM, Corning) and then at room temperature for 5 min with PI (20 µg/mL, EMD Millipore Corp., Burlington, MA, USA). Then, cells were washed with PBS and analysed in an inverted microscope (Axio Vert A1, Zeiss, Oberkochen, Germany)^26^.

## Supplementary Information

Supplementary Information.

## Data Availability

The datasets used and/or analysed during the current study available from the corresponding author on reasonable request.
