# Supplementary material for: Use of natural deep eutectic systems as new cryoprotectant agents in the vitrification of mammalian cells
Source: Sci Rep. 2022 May 16;12:8095. doi: 10.1038/s41598-022-12365-4 (PMC9110728; doi:10.1038/s41598-022-12365-4)
Supplement: Supplementary file 1 — Supplementary Information. [file 41598_2022_12365_MOESM1_ESM.docx]

**Use of Natural Deep Eutectic Systems as new cryoprotectant agents in the vitrification of mammalian cells**

**Acknowledgements**

This project has received funding from the European Research Council (ERC) under the European Union’s Horizon 2020 Research and Innovation Programme, under grant agreement No ERC-2016-CoG 725034. This work was also supported by the Associate Laboratory for Green Chemistry – LAQV, financed by national funds from FCT/MCTES (UID/QUI/50006/2019) and by FCT/MCTES through the project CryoDES (PTDC/EQU-EQU/29851/2017).

**Ana Rita Jesus^1^, Ana Rita C. Duarte^1^, Alexandre Paiva^1*^**

^1^ LAQV-REQUIMTE, Campus da Caparica, Monte da Caparica, 2825-149 Caparica, Portugal

* alexandre.paiva@fct.unl.pt

**A B**

**C D**

**E F**

**Figure S1.** Thermograms and POM images of **A.** BetSucProW, **B.** BetTreW, **C.** BetTreGlyW, **D.** **E.** DMSO and **F.** distilled water, between 40 ºC and 90 ºC. Physical changes at the T_c_ and T_m_, are also showed.

**A B**


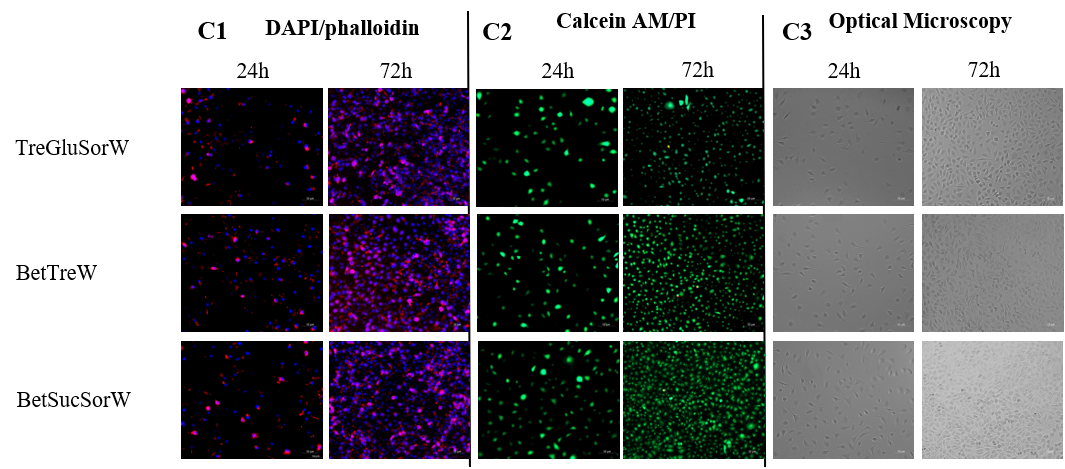


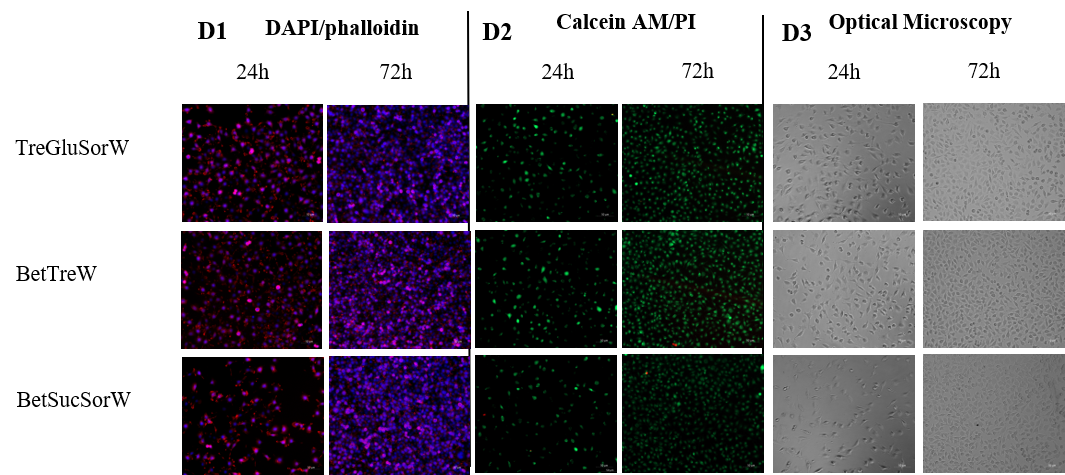


**Figure S2.** Post-thawing Cell Viability at different passages at different timepoints, for both cell lines. **A.** L929 P1; **B.** L929 P2; Data represent means ± SD (n = 3). Statistically significant differences were obtained by Turkey’s multiple comparison test and are represented in asterisks: * p < 0.05; ** p < 0.005, two-way ANOVA. The absence of asterisks means that there are no significant differences when compared with values of Me_2_SO at the corresponding time points. **C.** Post-thawing DAPI/Phalloidin assay (cells nuclei were stained blue by DAPI and F-actin filaments in red by phalloidin) (**C1**), Live/Dead fluorescence assay (living cells were stained green by calcein AM and dead cells red by PI) (**C2**) and Optical microscopy (**C3**) of L929 P1 cells at 24h and 72h. **D.** Post-thawing DAPI/Phalloidin assay (cells nuclei were stained blue by DAPI and F-actin filaments in red by phalloidin) (**D1**), Live/Dead fluorescence assay (living cells were stained green by calcein AM and dead cells red by PI) (**D2**) and Optical microscopy (**D3**) of L929 P2 cells at 24h and 72h.

**A B**


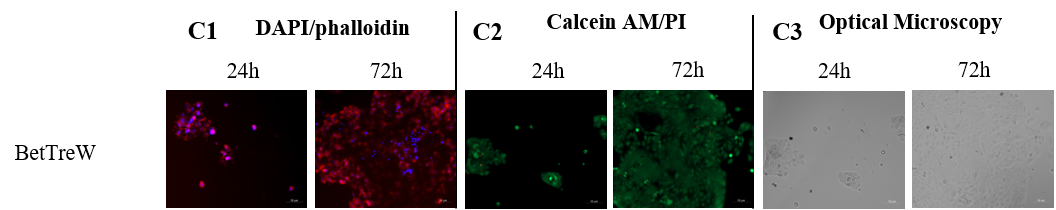


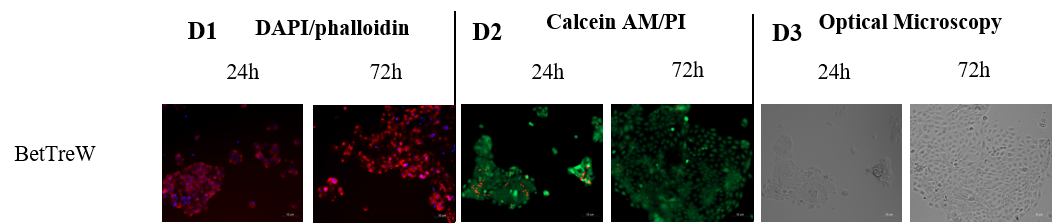


**Figure S3.** Post-thawing Cell Viability at different passages at different timepoints, for both cell lines. **A.** HacaT P1; **B.** HacaT P2; **C.** Post-thawing DAPI/Phalloidin assay (cells nuclei were stained blue by DAPI and F-actin filaments in red by phalloidin) (**C1**), Live/Dead fluorescence assay (living cells were stained green by calcein AM and dead cells red by PI) (**C2**) and Optical microscopy (**C3**) of HacaT P1 cells at 24h and 72h. **D.** Post-thawing DAPI/Phalloidin assay (cells nuclei were stained blue by DAPI and F-actin filaments in red by phalloidin) (**D1**), Live/Dead fluorescence assay (living cells were stained green by calcein AM and dead cells red by PI) (**D2**) and Optical microscopy (**D3**) of HacaT P2 cells at 24h and 72h.
